# Oral Hypoglycemic Drugs: Pathophysiological Basis of Their Mechanism of Action

**DOI:** 10.3390/ph3093005

**Published:** 2010-09-15

**Authors:** Bartolomeo Lorenzati, Chiara Zucco, Sara Miglietta, Federico Lamberti, Graziella Bruno

**Affiliations:** Department of Internal Medicine, University of Turin, Corso Dogliotti 14, 10126 Turin, Italy; E-Mails: lorebato@gmail.com (B.L.); chiara.zucco@gmail.it (C.Z.); sara.miglietta@libero.it (S.M.); federicolam@libero.it (F.L.)

**Keywords:** hypoglycemic drugs, sulfonylureas, thiazolidinediones, incretin mimetics

## Abstract

Type 2 diabetes is a syndrome characterized by relative insulin deficiency, insulin resistance and increased hepatic glucose output. Medications used to treat the disease are designed to correct one or more of these metabolic abnormalities. Current recommendations of the American Diabetes Association (ADA) and European Association for the Study of Diabetes (EASD) include diet and exercise as first-line therapy plus hypoglycemic drugs. Actually there are seven distinct classes of anti-hyperglicemic agents, each of them displaying unique pharmacologic properties. The aim of this review is to describe the pathophysiological basis of their mechanism of action, a necessary step to individualize treatment of diabetic people, taking into proper consideration potential benefits and secondary effects of drugs.

## 1. Introduction

Type 2 diabetes affects approximately 200 million people worldwide, including more than a quarter of elderly living in developed countries. Diet and exercise are first line treatments along with oral hypoglycaemic drugs to achieve the goal of improving glycaemic control and preventing both microvascular and macrovascular complications. There are seven distinct classes of hypoglycemic agents (Table 1): biguanides, sulfonylureas, meglitinides, thiazolidinediones, α-glucosidase inhibitors, incretin mimetics and DPP-4 inhibitors. Aim of this review is to describe the pathophysiological basis of their mechanism of action, to allow physicians to individualize treatment of diabetic people. 

**Table 1:** Oral hypoglycemic drugs and their class.

Drug class		Agent
**Biguanides**		Metformin
**Sulfonylureas**	*first generation*	Acetohexamide
		Chlorpropamide
		Tolazamide
		Tolbutamide
	*second generation*	Glibenclamide/Gliburide
		Glipizide
		Glimepiride
		Gliclazide
**Meglitinides**		Repaglinide
		Nateglinide
**Thiazolidinediones**		Rosiglitazone
		Pioglitazone
**α-Glucosidase inhibitors**		Acarbose
		Miglitol
**Incretin agonists**		Exenatide
		Liraglutide
**DPP-4 inhibitors**		Sitagliptin
		Vildagliptin
		Saxagliptin

## 2. Biguanides

Biguanides are old agents that work by reducing hepatic glucose output and, to a lesser extent, enhancing insulin sensitivity in hepatic and peripheral tissues (ie, antihyperglycemics, hepatic insulin sensitizers). Phenformin was taken off the market in the United States in the 1970s because of its risk of causing lactic acidosis and the associated mortality (rate of approximately 50%). In contrast, metformin has proved effective and safe [1]. It has been used in Europe for over thirty years, whereas in the United States it has been available since 1995. Metformin should be prescribed to all people with type 2 diabetes, unless contraindicated. Current recommendations of the American Diabetes Association (ADA) and European Association for the Study of Diabetes (EASD) include metformin, diet and exercise as first-line therapy for the treatment of patients with type 2 diabetes, irrespective of the presence of overweight status.

In contrast to sulfonylureas, metformin does not directly stimulate insulin secretion; its major effects are to increase insulin action and insulin-mediated glucose utilization in peripheral tissues (such as muscle and liver), particularly after meals, and to decrease hepatic glucose output. Moreover, it has an antilipolytic effect that lowers serum free fatty acid concentrations, thereby reducing substrate availability for gluconeogenesis [1]. Studies have shown that metformin is absorbed rapidly from the small intestine, with peak plasma concentrations obtained in two hours. Metformin reduces HbA1c levels by approximately 1.5 to 2.0 percent and fasting plasma glucose (FPG) levels by 50 to 70 mg per dL (2.8 to 3.9 mmol per L) [2,3]. Other effects include a reduction in plasma triglyceride levels and low-density lipoprotein (LDL) cholesterol levels. The most common side effects of metformin (10% of patients in the first days of treatment) are gastrointestinal, including a metallic taste in the mouth, mild anorexia, nausea, abdominal discomfort and soft bowel movements or diarrhea [4,5]. These symptoms are usually mild, transient, and reversible after dose reduction or discontinuation of the drug. To reduce these effects it is suggested to prescribe 500 mg once daily with the evening meal and, if tolerated, to add a second 500 mg dose with breakfast. The dose can be increased slowly (one tablet every one to two weeks) as necessary. The usual effective dose is 1,500 to 2,000 mg/day per day; the maximum dose of 2,550 mg/day (850 mg TID) provides only marginally better glycemic control and is often not tolerated due to gastrointestinal side effects. Extended release tablets are also available now, although patients who are doing well on immediate release metformin should probably continue with this preparation, as there is little, if any, additional benefit documented with the long-acting preparation. Combination tablets of metformin and sulfonylureas, thiazolidinediones, or dipeptidyl peptidase (DPP)-4 inhibitors are also available to increase patients’ compliance in the following phases of treatment. 

Recently, a potential protective effect with respect to neoplastic disease has been suggested by observational studies, but results need further confirmation. In one of these studies metformin monotherapy carried the lowest risk of cancer, whereas insulin therapy, compared with metformin, increased the risk of colorectal and pancreatic cancer, but did not influence the risk of breast or prostate cancer [5].

A rare problem (3 cases on 100,000 patients/years) is lactic acidosis, which may be fatal. The risk is much less with metformin than with another old biguanide, phenformin, which was withdrawn from use in the United States for this reason. Symptoms of lactic acidosis are nonspecific and may include anorexia, nausea, vomiting, abdominal pain, lethargy, hyperventilation, and hypotension. However, the incidence of lactic acidosis in metformin users appears to be very low [6]. In a review of 11,800 patients treated with metformin for a mean of about two years, only two patients developed lactic acidosis (incidence nine cases per 100,000 person-years of exposure) [3]. In a systematic review of 206 trials representing 48,000 patient-years in the metformin group and 38,000 patient-years in the comparator group, almost one-half of the studies allowed inclusion of patients with a serum creatinine above 1.5 mg/dL [133 mmol/L], and almost all of them allowed the inclusion of patients with at least one contraindication to metformin therapy [7]. Of all contraindications, metformin accumulation due to renal insufficiency is of greatest concern. As the serum creatinine alone may not be an accurate index of renal function in elderly patients or others with reduced muscle mass, GFR (Glomerular Filtration Rate) must be estimated. A GFR value less than 60 mL/min would be the approximate equivalent of the above serum creatinine cutoffs (1.4 mg/dL [124 micromol/L] in women and 1.5 mg/dL [133 micromol/L] in men) and metformin should be discontinued. It is very relevant to remember that patients who are about to receive intravenous iodinated contrast material (with potential for contrast-induced renal failure) or undergo a surgical procedure (with potential compromise of circulation) should have metformin stopped until stable renal function can be established (normal urine output, normal serum creatinine and no physical exam evidence of fluid overload or circulatory compromise). The pathogenesis of metformin-associated lactic acidosis is not completely understood. It is known that metformin impairs lactate clearance of the liver through the inhibition of complex I of the mitochondrial respiratory chain [7]. Especially when metformin levels are high, oxidative phosphorylation is reduced and aerobic metabolism switches to anaerobic metabolism [8]. Although increased lactic acid production may be induced by haemodynamic instability and/or tissue hypoxia associated with severe metformin overdose or any underlying unstable cardiovascular or respiratory condition, lactic acidosis is predominantely due to a lack of lactate's clearance than to an increased production. When used according to current prescribing recommendations, however, the risk of metformin-induced lactic acidosis is close to zero [6]. Moreover, despite the appreciable number of contraindications, the incidence of metformin-induced lactic acidosis is not increasing. These findings have led someone [9] to recommend a revaluation of the contraindications to metformin therapy. 

## 3. Sulfonylureas

Sulfonylureas were the mainstay of antidiabetic therapy since the early 1950s. Following the release of the University Group Diabetes Program (UGDP) study, which implicated tolbutamide in increased mortality due to cardiovascular events, the use of the first generation sulfonylureas (acetohexamide, chlorpropamide, tolbutamide and tolazamide) quickly fell out of favour. In contrast, the second-generation sulfonylureas (glipizide, gliclazide, glibenclamide, called also glyburide) and glimepiride are widely employed worldwide. They work by stimulating insulin release from the insulin secreting ß-cells located in the pancreas [10] and may slightly improve insulin resistance in peripheral target tissues (muscle, fat) [11]. Their receptor is a component of the ATP-dependent potassium channel in the pancreatic β-cells; the binding leads to inhibition of these channels, which alters the resting potential of the cell, leading to calcium influx and stimulation of insulin secretion. The net effect is increased responsiveness of β-cells to both glucose and non-glucose secretagogues, resulting in more insulin being released at all blood glucose concentrations. Due to their mechanism of action, studies have suggested concern with respect to patients with acute myocardial infarction. In the DIGAMI (Diabetes Mellitus, Insulin Glucose Infusion in Acute Myocardial Infarction) trial, patients with diabetes mellitus and acute myocardial infarction were randomly allocated to standard treatment plus insulin-glucose infusion for at least 24 hours followed by multidose insulin treatment or standard treatment (controls) [12]. The patients with the poorest outcome were those treated with a sulfonylurea at the time of the myocardial infarction. A retrospective observational cohort study performed on 568 outpatients (282 women, 286 men) with type 2 diabetes treated with either glibenclamide (n = 378) or gliclazide (n = 190) suggested that treatment with glibenclamide could be associated with higher mortality for cardiovascular diseases, in comparison with gliclazide, but only in patients with previously known ischemic heart disease [13]. More recently, the ADVANCE trial, an intensive glucose-control strategy involving gliclazide (modified release), and other drugs asrequired, reduced incidence of the combined primary outcome of major macrovascular or microvascular events. The main contributor to the 10% relative reduction in the primary outcome with intensive control as compared with standard control was a 21% relative reduction in the risk of new or worsening nephropathy. There was no evidence of a reduction in macrovascular events. Intensive glucose control was associated with an increased risk of severe hypoglycemia and an increased rate of hospitalization, as compared with standard control [15]. 

Sulfonylureas differ mainly in their potency and duration of action. The second generation have a potency that allows them to be given in much lower doses than the first generation. Drugs with longer half-lives (particularly chlorpropamide, glibenclamide and glimepiride) can be given once daily. They cause greater suppression of overnight hepatic glucose output, thereby lowering fasting blood glucose concentrations more; these benefits, however, may be counterbalanced by an increased risk of hypoglycemia. Sulfonylureas can be used as monotherapy, or in combination with other oral hypoglycemic drugs or insulin. This class reduces glycosylated hemoglobin A1c (HbA1c) levels by 0.8 to 2.0 percent and FPG concentrations by 60 to 70 mg per dL (3.3 to 3.9 mmol per L), with the greatest reductions observed in patients with the highest FPG concentrations at the initiation of therapy [16] . All sulfonylureas have been associated with weight gain, unless the diabetic diet and exercise program are followed, and thus are not suggested as first choice for obese patients. In the UKPDS Study the increment in weight was of 2.6 Kg for those assigned to chlorpropamide and 1.7 Kg for those assigned to glibenclamide. They are most likely to be effective in patients whose weight is normal or slightly increased. The choice of sulfonylurea is primarily dependent upon cost and availability, because their efficacy against microvascular and cardiovascular complications is similar [17]. Given the relatively high incidence of hypoglycemia in patients taking glyburide or chlorpropamide, shorter acting drugs should be preferred, especially in elderly patients [18]. 

## 4. Meglitinides

The meglitinides, repaglinide and nateglinide, are short-acting glucose-lowering drugs for therapy of patients with type 2 diabetes alone or in combination with metformin. They were designed to achieve more physiologic insulin release and less risk for hypoglycemia. They are structurally different than sulfonylureas but their mechanism of action closely resembles that of sulfonylureas (they act by regulating ATP-dependent potassium channels in pancreatic beta cells), because they stimulate the release of insulin from the pancreatic beta cells through a different binding site on the “sulfonylurea receptor” [19]. Moreover, meglitinides have different half-life compared to sulfonylureas. Because of the short onset of action of the meglitinides (15 to 30 minutes), patients should be instructed to administer a dose immediately before a meal. The meglitinides can be used as monotherapy, or in combination with other oral hypoglycemic drugs like metformin, resulting in superior glycemic control than with either agent used as monotherapy. Their clinical efficacy is similar to that of the sulfonylureas. Some potential advantages of this class of agents include a greater decrease in postprandial glucose and a decreased risk of hypoglycemia. A meta-analysis of 15 trials to assess the efficacy of meglitinides compared with placebo, metformin, or in combination with insulin reported that both meglitinides reduced HbA1C values, with a greater reduction in HbA1C occurring in those receiving repaglinide compared with nateglinide (0.1 to 2.1 *versus* 0.2 to 0.6 percentage point reduction, respectively) [20]. Repaglinide had similar efficacy in reducing HbA1C values as metformin, whereas nateglinide was similar or slightly less effective. Repaglinide has shown similar effects on HbA1c and FPG levels when compared with glyburide, 0.5 to 2 percent and 65 to 75 mg per dL (3.6 to 4.2 mmol per L), respectively. The recommended starting dose of repaglinide is 0.5 mg before each meal for patients who have not previously taken oral hypoglycemic drugs. Repaglinide can be titrated to a dosage of 4 mg before each meal (maximum dosage of 16 mg per day). Nateglinide can be titrated to a dosage of 120 mg taken immediately before each meal. At least one week should be allowed between dosage adjustments to adequately assess blood glucose response. This unique dosing regimen may allow greater flexibility for patients who have difficulty maintaining a regular meal schedule. Hypoglycemia is the most common adverse effect. Nateglinide is hepatically metabolized, with renal excretion of active metabolites. As repaglinide is principally metabolized by the liver, with less than 10 percent renally excreted, it is the drug of choice in patient with impaired renal function [21]. Dose adjustments with this agent do not appear to be necessary in patients with renal insufficiency.

There are no long-term studies of meglitinides assessing cardiovascular outcomes or mortality in patiens treated with his class of drugs. Whether meglitinides are associated with poorer outcomes after a myocardial infarction is not known. However, since its mode of action is similar to sulfonylureas, the same concern exists.

## 5. Thiazolidinediones

During the last decade a new class of drugs have been available for treatment of type 2 diabetes: the thiazolidinediones (troglitazone, rosiglitazone and pioglitazone). Actually only two thiazolidinediones (rosiglitazone and pioglitazone) are currently marketed. The majority of data reporting the efficacy of this class comes from studies with troglitazone, results from more recent studies with the newer agents (rosiglitazone and pioglitazone) demonstrating similar properties, although their mechanism of action is not fully understood [22].

Thiazolinediones improve glycemia reducing insulin resistance and preserving pancreatic beta-cell function with different mechanism of action; as example, the predominant effect of metformin is to inhibit hepatic glucose production, whereas thiazolidinediones act mainly by improving peripheral uptake and utilization of glucose in muscle and fat, finally decreasing liver glucose production [23]. In human adipocytes, rosiglitazone treatment increases expression of genes involved in promoting lipid storage and decreases expression of genes associated with inflammation, such IL-6 [24]. These drugs activate one or more peroxisome proliferator-activated receptors (PPARs), which regulate gene expression in response to ligand binding [25]. PPAR-γ is found predominantly in adipose tissue, pancreatic beta-cells, vascular endothelium, and macrophages; its concentration is also increased in skeletal muscle of obese and diabetic patients [26]. PPAR-α is expressed mostly in liver, heart, skeletal muscle, and vascular walls. It is interesting to note that various thiazolidinediones have differential effects on PPAR-γ and PPAR-α. Troglitazone and rosiglitazone are purely PPAR-γ agonists, while pioglitazone also exerts some PPAR-α effects. This may account for different effects within this class of drugs. In adipose tissue the insulin-sensitizing effect may be related to the production of adipokines via PPAR-gamma activation [27]. 

Several new thiazolidinediones are being investigated as "dual PPAR agonists," with the hope to treat both hyperglycemia and hyperlipidemia. One dual agent, muraglitazar, received initial FDA approval but data reinterpretation has led to concerns about cardiac safety (increased incidence of a composite outcome of death, MI, stroke, transient ischemic attack, or heart failure) [28]. Another investigational dual PPAR agonist, aleglitazar, performed favorably in a 16-week dose ranging phase II trial (SYNCHRONY); it improved HbA1C, triglycerides, LDL, and HDL cholesterol compared with placebo. Larger, long-term trials with cardiovascular outcomes are planned [29].

Pioglitazone and rosiglitazone similarly improve blood glucose level; their efficacy is comparable to metformin as monotherapy. However, thiazolidinediones are not generally indicated over metformin for initial therapy of type 2 diabetes as they are among the most expensive oral agents. Thiazolidinediones may have antiinflammatory, antithrombotic, and antiatherogenic properties. Although they seem to improve a number of cardiovascular risk factors and their surrogate cardiovascular endpoints (dyslipidemia, endothelial function, vascular smooth muscle proliferation, markers of inflammation [30], carotid intima media thickness [31], vascular reactivity and progression of atherosclerosis on coronary intravascular ultrasound [32,33]), both drugs enhance incidence of heart failure. However, they might have different effects on ischemic outcomes [34,35]. Some studies and meta-analyses have suggested that rosiglitazone increase the risk of myocardial infarction (MI) [36,37], not confirmed by other studies [38,39], whereas pioglitazone may not have the same cardiovascular risk profile than rosiglitazone: in a meta-analysis of 19 trials of pioglitazone MIs occurred in 131 (1.5 percent) patients in the pioglitazone group and 159 (2.0 percent) in the comparator group (placebo, metformin, sulfonylurea, rosiglitazone) (HR 0.81, 95% CI 0.64–1.02). The primary composite endpoint of death, nonfatal MI, or nonfatal stroke occurred in 4.4 and 5.7 percent of patients in pioglitazone and control groups, respectively (HR 0.82, 95% CI 0.72–0.94) [36]. It should be noted, however, that neither studies nor meta-analyses were designed to explore cardiovascular outcomes, therefore misclassification could have affected the data.

The RECORD study was designed to evaluate the effect of rosiglitazone on cardiovascular events and mortality in 4,447 patients from Europe and Australasia. Subjects who failed metformin or sulfonylurea monotherapy (HbA1C > 7 percent) were randomly assigned to addition of rosiglitazone, metformin (if initially on sulfonylurea), or sulfonylurea (if initially on metformin) [40]. In the final analysis (mean 5.5 years of follow-up), 321 and 323 subjects in the rosiglitazone and in the control groups, respectively, experienced the primary endpoint (cardiovascular hospitalization or cardiovascular death). The lower than expected event rate and the higher drop-out rate (18 percent of all subjects) decreased the power of the analysis to assess the primary outcome, except for heart failure. There was an increased risk of fatal and non-fatal heart failure, occurring in 61 subjects assigned to rosiglitazone compared with 29 in the control group (HR 2.10, 95% CI 1.35–3.27). The effect of rosiglitazone on myocardial infarction was inconclusive (HR for rosiglitazone compared with the active comparators for fatal and non-fatal MI 1.14, 95% CI 0.80-1.63) due to the small number of events [41].

The PROspective pioglitAzone Clinical Trial In macroVascular Events (PROACTIVE) trial was designed to evaluate the effect of pioglitazone on cardiovascular events and mortality in 5,238 patients at high risk for macrovascular complications (prior MI, stroke, CABG, acute coronary syndrome or symptomatic peripheral arterial disease). The study was stopped prematurely because of a significative decrease in the "main" secondary composite end point of all-cause mortality, MI (excluding silent MI), or stroke in pioglitazone group (HR 0.84, 95% CI 0.72–0.98). However, there was a not significant impact on the predefined primary outcome of the study: composite of all cause mortality, nonfatal MI and silent MI, stroke, acute coronary syndrome, surgical intervention on coronary or leg arteries or leg amputation. Reports of HF were higher (16.0 *versus* 11.5 percent) in the pioglitazone group [42]. In conclusion, whereas the cardiovascular benefit/risk ratio of pioglitazone is evident, results of the meta-analyses suggest increased caution with rosiglitazone, in particular until additional data either dispute or confirm its cardiotoxic effects.

All thiazolidinediones can cause weight gain due in part by fluid retention (peripheral edema occurs in 4% to 6 %), but also from the proliferation of new adipocytes. Thiazolidinediones act by binding to and activating PPAR-γ: along the nephron, PPAR-γ is most abundant in the collecting tubules, and the fluid retention with thiazolidinediones appears to result from PPAR-γ stimulation of sodium reabsorption by sodium channels (called the epithelial sodium channel) in the luminal membrane of the collecting tubule cells [43]. This effect is mediated by increased expression of the gamma subunit of the sodium channel gene mRNA. Macular edema has been reported in patients taking thiazolidinediones: patients also at risk for peripheral edema seem to be at greatest risk .

Thiazolidinediones have an effect on bone metabolism through the activation of peroxisome proliferator activated receptor, so a relationship between their assumption and a increased risk of fractures has been supposed. Some informations come from A Diabetes Outcome Progression Trial (ADOPT), in which women in the rosiglitazone group had a major rate of bone fractures than women treated with metformin or glyburide; limitations are that confounding variables, like pre-existing osteoporosis, were not taken into consideration and that fractures were self reported by the partecipants and were not screened for; so adjunctive studies are necessary to investigate this relationship [44].

## 6. α-Glucosidase Inhibitors

α-Glucosidase inhibitors include acarbose and miglitol. They act on α-glucosidase, an enzyme found in brush border cells of small intestine, cleaving more complex carbohydrates into sugars. α-Glucosidase inhibits the breakdown and absorption of carbohydrates (dextrins, maltose, sucrose and starch; no effect on glucose); their largest impact is on postprandial hyperglycemia and their effect on FPG levels is modest. They have been associated with a reduction in HbA1c by 0.7 to 1.0 percent and FPG levels by 35 to 40 mg per dL (1.9 to 2.2 mmol per L) [45].

These agents are thus most useful in patients who have mild FPG elevations or in patients with predominant postprandial hyperglycemia. However, the main side effects of α-glucosidase inhibitors are flatulence, abdominal discomfort, bloating and diarrhea, which reduce compliance in treated patients. As for metformin, patients should be instructed to take this medication with food, starting with the lowest effective dose and titrated slowly over intervals of two to four week. Although hypoglycemia is not typically associated with monotherapy with α-glucosidase inhibitors, it can occur in combination with other drugs; it is important, to inform patients that the traditional treatment for hypoglycemia may be blocked during treatment with α-glucosidase inhibitors and only glucose should be consumed in this condition. 

## 7. Incretin Mimetics and Incretin Enhancers Drugs

Incretins (Glucagon Like Peptide-1 and Glucose Dependent Insulinotropic Polypeptide) are enteroendocrine hormones released into bloodstream from L and K cells dispersed throughout the gastrointestinal tract [46]. 

GLP-1 is secreted in response to nutrients and its levels are decreased in type 2 diabetes; it acts stimulating glucose-dependent insulin release from the pancreatic islets and this is the major advantage over sulfonylureas, as it prevents hypoglycaemia [47]. It also slows gastric emptying, inhibit inappropriate postmeal glucagon release, and reduce food intake. Owing in part to the effects of GLP-1 on slowed gastric emptying and its well recognized side-effects of nausea and vomiting, therapy with GLP-1 and its analogs is associated with weight loss. GLP-1 exhibits a short half-life of one to two minutes due to N-terminal degradation by the enzyme dipeptidyl peptidase IV (DPP-IV) [48]. Research has focused on GLP-1 like analogs that are resistant to DPP-IV degradation and on agents that increase GLP-1 via inhibition of DPP-IV. Experimental studies have suggested that GLP-1 stimulate proliferation of developed β-cells and inhibits B-cell apoptosis [49], suggesting a potential role of incretin-mimetics *in vivo* in limiting β-cells dysfunction, which typically occurs in people with type 2 diabetes. GLP-1 might have beneficial effects on myocardial function: improves myocardial contractility, improves glucose uptake in normal and postischemic rat hearts, induces an endothelial-dependent reduction in vascular tone of rat lungs. 

Exendin-4 is a naturally occurring peptide identified in the saliva of Gila monster (Heloderma suspectum); it has 53% homology with human GLP-1 amino acid sequence; his molecular structure makes it considerably more resistant than active GLP-1 to degradation by DPP-4. 

Exenatide, a synthetic version of exendin-4, is a 39–amino acid peptide incretin mimetic that exhibits glucose regulatory activities similar to those observed with human GLP-1 but with increased resistance to deactivation by DPP-4. Exenatide binds GLP-1 receptor, stimulates glucose-dependent insulin secretion, suppresses glucagon secretion, slows gastric emptying and reduces food intake [50]. More interestingly, exenatide appears to have beneficial effects on β-cell function: indeed, in addition to glucose-dependent insulin stimulation, exenatide normalizes the loss of first-phase insulin secretion as well as hypersecretion of glucagon from α-cells, thereby reducing hepatic glucose production in the postprandial state [51]. Clinical guidelines suggests that it is indicated as an adjunctive therapy to improve glycaemic control in patients with type 2 diabetes who are already receiving metformin, a sulfonylurea, or both, but continue to have suboptimal glycaemic control [52]. Exenatide should be initiated at 5 mcg per dose, administered twice daily at any time within the 60-minute period before the morning and evening meals. Exenatide lowers glycosylated hemoglobin (HbA1C) levels by 0.4% to 0.9%, with weight reductions of 0.9 to 3.1 kg [53]. Reductions of systolic blood pressure ( 3.4–3.7 mm Hg) and diastolic blood pressure (0.8–2.3mm Hg) have also been reported [54]. 

Randomized open-label trials comparing exenatide with insulin glargine or biphasic insulin aspart, combined with metformin and a sulfonylurea in patients with poorly controlled type 2 diabetes, demonstrated similar HbA1C reductions of -0.89 to -1.1% in the exenatide and insulin groups. The target level was reached in only 21.6% of the subjects randomly assigned to glargine, and the average dose used (25 units) was substantially lower than in most studies. The relatively unaggressive insulin therapy may have balanced overall glycemic control in favor of exenatide. The exenatide groups exhibited weight loss as opposed to weight gain and improved postprandial glucose control compared with either insulin group, and less nocturnal hypoglycemia compared with insulin glargine [55].

Exenatide has a relatively short mean terminal half-life of 2.4 hours. The predominant route of elimination is via glomerular filtration with subsequent proteolytic degradation; consequently, exenatide is not recommended for use in patients with severe renal impairment (GFR<30 mL/minute) or end-stage renal disease. In patients with moderate renal impairment (creatinine clearance 30 to 50 mL/min), monitoring of serum creatinine is warranted when initiating therapy and after the usual dose increase from 5 to 10 mcg. Nausea is the most commonly (45-51%; placebo 7-23%) reported adverse effect of exenatide; it is more obvious in fasting subjects and is probably a direct central effect. Other adverse events, occurring in 10%, of patients are diarrhea (12.8%), and vomiting (12.8%). Weight loss is common, dose dependent, and was not solely due to incidence of nausea, although treated patients with nausea lose substantially more weight than patients without nausea. Hypoglycaemic events are mainly observed when combinations are used: in 20–30% of patients treated in combination with a sulfonylurea and in 13% of patients treated in combination with a thiazolidinedione [56]. It is relevant to notice that hypoglycaemia is not observed in people treated with incretion mimetics when this class of drugs is given together with metformin. Some patients (27–49%) treated with exenatide developed antibodies; and glucose-lowering efficacy appeared to be attenuated in patients with high-titer anti-exenatide antibodies (HbA1C reduction of 1.4%) compared with those with low-titer antibodies (reduction of 2.0%) or in those tested negative (reduction of 1.9%).

There have been 36 postmarketing reports of acute pancreatitis (necrotizing or hemorrhagic) in patients taking exenatide. The overall reported rate for pancreatitis in exenatide users is 1 in 3,000 and for the more severe necrotizing or hemorrhagic forms, less than 1 in 10,000, which is similar to the background rate in patients with diabetes mellitus [57].

Liraglutide is a human GLP-1 analog that has been modified to non-covalently bind to serum albumin through a lipid side chain, resulting in slower degradation and allowing for once-daily subcutaneous dosing; in Europe it is used in combination with metformin and/or a sulfonylurea or in combination with metformin and a thiazolidinedione. It causes suppression of glucagon secretion, glucose-dependent insulin release and delayed gastric emptying [58]. Liraglutide has been associated with HbA1C reductions of 0.6% to 1.5% and mean body weight reductions of 1.0 to 3.2 kg; it improves factors associated with cardiovascular disease like systolic blood pressure, plasminogen activator inhibitor-1 or natriuretic peptide levels. With respect to exenatide it has a significantly lower rate of hypoglycemia (1.9 *vs*. 2.3 events per patient-year; P = 0.0131); gastrointestinal side effects are less important and tend to diminuish over time. There is a low incidence of antiliraglutide antibodies (9–13%) that did not appear to affect the glucose-lowering effect [59]. In animal studies, liraglutide was associated with benign and malignant thyroid C-cell tumors. The potential effect of liraglutide on thyroid C-cells in humans requires further investigation. Until such data are available, liraglutide is not recommended for use in patients with a family history of medullary thyroid cancer or multiple endocrine neoplasia 2A.

DPP-4, described in 1966 and also known as CD26 regarding its activity in immune system, is a 110 kDa plasma membrane-spanning cell surface glycoprotein ectopeptidase, ubiquitously expressed in tissues such as liver, lung, kidney, intestinal brush-border membranes, lymphocytes, and endothelial cells. DPP-4 rapidly degrades and inactivates GLP-1, GIP, and other peptides *in vivo* via cleavage of N-terminal two amino acids. Inhibition of this enzyme leads to an increase in circulating endogenous GLP-1 and GIP levels; so that DPP-4 inhibitors are not incretin mimetics, but incretin enhancers. Unlike other GLP-1 based therapies, can be administered orally. 

Sitagliptin, vildagliptin and saxagliptin are DPP-IV inhibitors that are approved as initial pharmacologic therapy for the treatment of type 2 diabetes; as a second agent in those who do not respond to a single agent, such as a sulfonylurea, metformin or a thiazolidinedione; and as a third agent when dual therapy with metformin and a sulfonylurea does not provide adequate glycemic control. The usual dose of sitagliptin is 100 mg once daily, with reduction to 50 mg for moderate to severe renal insufficiency (GFR <30 to 50 mL/min) and 25 mg for severe renal insufficiency (<30 mL/min) [60]. The usual dose of saxagliptin is 2.5 or 5 mg once daily, with the 2.5 mg dose recommended for patients with moderate to severe chronic kidney disease (GFR ≤ 50 mL/min) and for patients taking strong cytochrome P450 3A4/5 inhibitors (e.g., ketoconazole).

DPP-4 inhibitors mimic the therapeutic effects of incretin mimetics including stimulation of insulin secretion, inhibition of glucagon secretion, possibly preservation of β-cell mass and inhibition of apoptosis. These drugs display quite similar efficacy in lowering HbA1C (≤1% reduction) compared with other antihyperglycemic agents, but they are weight neutral and have a low potential for hypoglycaemia when used as monotherapy. One safety concern involves the potential of DPP-4 inhibitors to interfere with immune functions: a meta-analysis of pooled clinical trial data for sitagliptin and vildagliptin indicates an increased risk for infection (nasopharyngitis [61] and urinary tract infection) and headache. Other adverse effects occurring with more frequency in sitagliptin—treated patients *versus* those receiving placebo include, back pain, osteoarthritis, and extremities pain [62].

## 8. Conclusions

The first line of treatment are lifestyle modifications and metformin. If metformin alone cannot achieve a good glycemic control or it is not tolerated or is contraindicated, a second drug selected among the sulfonylureas, thiazolinediones, incretin mimetics and incretin enhancer drugs must be used. What is particularly relevant, anyway, is to avoid therapeutic inertia, thus therapy should be modified as soon as possible to keep glycemic control HbA1c at about 7%. In this second step, various factors such risk of hypoglycemia, comorbidities, age of patients, presence of diabetic complications and cost of treatment must be properly considered to individualize treatment. 

## Abbreviations

ADA: American Diabetes Association
EASD: European Association for the Study of Diabetes
DPP-4: dipeptidyl peptidase
GFR: Glomerular Filtration Rate
FPG: fasting plasma glucose
HR: Hazard ratio
HbA1C: glycosylated haemoglobin
PPAR: peroxisome proliferator-activated receptor
GLP-1: Glucagon Like Peptide-1
GIP: Glucose Dependent Insulinotropic Polypeptide
MI: myocardial infarction
CABG: coronary artery bypass graft
